# Validation of a Hypothesis: Colonization of Black Smokers by Hyperthermophilic Microorganisms

**DOI:** 10.3389/fmicb.2018.00524

**Published:** 2018-03-21

**Authors:** Reinhard Wirth, Manja Luckner, Gerhard Wanner

**Affiliations:** ^1^Faculty of Biology, Archaea Centre, University of Regensburg, Regensburg, Germany; ^2^Department of Biology I, Ludwig-Maximilians-University, Munich, Germany

**Keywords:** black smoker, colonization, hyperthermophilic microorganisms: motility, adhesion, black smoker material, high temperature light microscopy, electron microscopy

## Abstract

Newly erupted black smokers (hydrothermal vent chimneys) are sterile during their formation, but house hyperthermophilic microorganisms in substantial amounts in later stages. No direct experimental data exist by which mechanisms hyperthermophiles colonize newly erupted black smokers, but a scenario was proposed recently how this might happen. Here we combine high temperature light microscopy with electron microscopy to show that two hyperthermophilic Archaea, namely *Pyrococcus furiosus and Methanocaldococcus villosus* are able to adhere onto authentic black smoker material (BSM). We especially are able to directly observe the adhesion process via video recordings taken at high temperatures. These data validate the hypothesis that hyperthermophiles are transferred by serendipitous water currents to the outside of newly formed black smokers and react within seconds to the there prevailing high temperatures by very fast movements. They scan the surface of the hydrothermal chimneys via a much slower zigzag seek-movement and adhere via their flagella at a suitable place, building up biofilms.

## Introduction

Upon their formation black smokers have to be sterile because the effluents—which come into contact with rocks heated by an underlying magma chamber—have temperatures of 300–400°C. The metals and sulfides which are dissolved in these effluents precipitate upon their contact with the surrounding (ca. 2°C) sea water to form hydrothermal vent chimneys of different sizes and wall thickness (see e.g., Haymon, 1983; Holden et al., 2012 and Dick et al., 2013 for details). Already the very first report on hydrothermal vents (Corliss et al., 1979) states that “*the high concentration of bacteria in water flowing from the vents suggests that they live to some depth in the rock mass, lining fractures and fissures, and that they may significantly influence the chemistry of the system.”* Water temperatures of these vents were 17°C at maximum, but this study was seminal for the discovery of black smokers, because it was argued already then from the chemistry data of such hydrothermal fluids that: “*the* [warm effluent] *seawater last equilibrated with the rocks at a temperature of about 300*°*C”* and speculated that: “*hot springs at greater than 100*°*C may be common”* in submarine hydrothermal systems. The concentrations of microorganisms in the vicinity of hydrothermal vents can be as high as 10^9^ per ml (Corliss et al., 1979), but also be substantial lower. Besides these “Galapagos type vents” another type of vents, the “sulfide-mound hot water vents” were described shortly thereafter (Spiess et al., 1980) and named black smokers. These black chimneys were reported to be free of organisms, but in this geological study inspection was only for (eukaryotic) organisms clearly visible by naked eye. No data for microbial life at/in black smokers have been reported by Spiess et al. (1980).

Because of the very high temperature of the effluent waters (300–400°C) newly formed hydrothermal vent chimneys cannot contain living microorganisms—colonization of black smokers has to be from the outside. Microbial cell counts for a black smoker chimney called Finn have been determined by epifluorescence to be in the range of 10^5^–10^6^ (Z1), ~10^8^ (Z2), ~10^7^ (Z3), and ~10^5^ (Z4); Z1 to Z4 were the exterior to interior zones of Finn (Schrenk et al., 2003). A detailed study on the distribution of Archaea in a black smoker (Takai et al., 2001) revealed values in the range of 10^7^–10^5^ cells per g (wet weight). Various experimental approaches like cultivation assays, 16S-rRNA-gene analyses, the combination of both, but also FISH experiments indicate that various hyperthermophiles exist in samples of black smokers. These microorganisms include Aquificales, Archaeoglobi, Desulfurococcales, Epsilonproteobacteria, Ignicoccales, Methanococcales, Thermococcales, and others (see Wirth, 2017 for details). Since black smokers are (at least to some degree) porous structures also cold background seawater might infiltrate them; therefore, it is not surprising to detect also mesophilic microorganisms at least in some parts of the vent chimneys. Even if one takes care to sample only “hot material,” low temperature sea water will be present in such samples (due to the sampling procedures used), adding to the observation that also mesophilic microorganisms can be identified in “hot samples.”

Recently a scenario was formulated (Wirth, 2017) to explain how hyperthermophiles—present in a kind of dormant state in low temperature deep-sea water—can colonize black smokers. It was based mainly on experimental data from the author's labs; direct observations how hyperthermophiles do interact with authentic black smoker material (BSM), however is missing. Here we show that (at least) two hyperthermophilic Archaea can bind to BSM using light microscopy, electron microscopy, and especially high temperature light microscopic video recordings, thereby validating this hypothesis.

## Materials and methods

### Growth of cells and high temperature video light microscopy

The two model organisms for hyperthermophilic Archaea, *Pyrococcus furiosus* (Fiala and Stetter, 1986) and *Methanocaldococcus villosus* (Bellack et al., 2011) were grown at 95°C in ½ SME medium or at 80°C in MJ medium, respectively. For the actual binding assays overnight cultures were diluted 1:100 in 10 ml of the respective medium and the suspension of solids added in a 1:10 dilution. Samples were withdrawn with a syringe after appropriate times of incubation (3 h for *P. furiosus* and 2 h for *M. villosus*) and analyzed by light microscopy for adhesion.

Positive samples immediately were analyzed by high temperature video light microscopy in the temperature gradient forming device (TGFD; Mora et al., 2014) as described earlier (Herzog and Wirth, 2012). This in short included: collection of a ~0.5 ml sample into an Eppendorf-Cup into which immediately rectangular glass capillaries were placed which filled themselves via capillary forces within 20 s. The capillaries were removed from the Eppendorf-Cup, liquid was dried from the glass surface by paper-wipes and the ends of the capillaries closed by three layers of super-glue. The whole procedure took <3 min and therefore could be done aerobically at room-temperature. Video analyses were done at the respective growth temperatures and movies recorded for a maximum time of 3.5 min at a final magnification of 400-fold; in some cases a secondary magnification of 2 was used for recording videos. The very same samples used for video recordings were in addition used to prepare samples for scanning electron microscopy (SEM).

### Solids used for adhesion studies

As a first proxy for BSM we used “lava” (= porous, volcanic glass of basaltic origin) to establish the conditions to be used in further experiments. A commercial lava sample, sold to promote plant growth by addition into potting soil, turned out to inhibit growth of both Archaea and was not further used. Therefore, small pieces of lava collected in March 2017 in Iceland (at the beach of lake Mývatn: 65° 36′ 0″ N, 17° 0′ 36″ W) were ground in a mortar to a size below 0.1 mm and washed extensively with water. This material was suspended in ½ SME or MJ medium at a final concentration of 2.5% (w/v), gassed with N_2_/CO_2_ and autoclaved. For adhesion studies this lava preparation was added to growing cultures at a final dilution of 1:10.

The authentic BSM sample “MEX13C”—a small black smoker, ca. 10 × 30 cm—was collected on January, 13 of 2008 during cruise AT15-28 (Fix08) with the deep-sea submersible Alvin (dive # AD 4398) at the East Pacific Rise (southwest of Mexico: 9° 50.39936 N; 104° 17.48796 W) at a depth of 2,507 m. After return to surface the collected black smoker (stored during the ascent in Alvin's “Biobox”) was broken into ca. 1 cm pieces, fine particles were scraped off with a razor blade and the combined materials placed into a 100 ml Schott bottle; sterile filtered seawater (0.22 μm) was added to a final volume of 100 ml in the bottle, which was sealed with a rubber stopper (20 mm height). The sample was reduced by addition of 1 ml of 2.5% sodium-sulfide solution and stored at 4°C. In 2013 three solid pieces of BSM ca. 1 cm^3^ each, together with ca. 3 cm^3^ of fines were removed from the original sample in an anaerobic chamber, placed in a sterilized mortar and ground under anaerobic conditions with a piston to result in fine ground BSM with a maximal particle size of <1 mm. These solids again were transferred into a Schott bottle; sterilized ½ SME was added, the sample reduced with 0.025% sodium-sulfide (end concentration) and stored at 4°C. For adhesion studies this preparation was added to growing cultures in a final 1:10 dilution.

### Sample preparation and scanning electron microscopy

Samples for SEM were prepared by collecting 5 ml aliquots of the binding assays analyzed by light microscopy in glass tubes and letting coarse particles sediment for 30 s. Thereafter 4.5 ml of the sample was transferred into a centrifuge tube and particles sedimented by centrifugation at 1,000 g for 1 min. The supernatant was removed, the very loose pellet suspended in 5 ml medium +2.5% glutaraldehyde, and the procedure repeated. The final pellet was suspended in 0.25 ml medium +2.5% glutaraldehyde, stored at 4°C and used within 48 h for sample preparation for SEM.

Sample preparation and SEM was done by to procedures established previously in our labs (Rachel et al., 2010; Jogler et al., 2011). In short these included: dehydration with a graded series of acetone solutions; critical point drying from liquid CO_2_; mounting on stabs with conductive tabs; sputter coating with platinum (3–5 nm).

For ultrastructural analysis specimens were examined with a Zeiss AURIGA^®^ high-resolution field-emission scanning electron microscope operated at 1–1.5 keV. For energy-dispersive X-ray (EDX) analysis (point analysis and EDX mapping) a Bruker QUANTAX double detector system (2 × 30 mm^2^ XFlash^©^) was used at a working distance of 6 mm and an accelerating voltage of 15 kV.

## Results

### Binding experiments using lava and BSM

Our first experiments used commercial lava, intended to be used as additive for plant soil. Adhesion was observed to these solids, but the material also inhibited growth of the two Archaea analyzed. We therefore used for establishment of best experimental conditions a freshly collected sample of lava, which after extensive washings with water did not influence the growth of both Archaea.

These initial experiments indicated that binding of cells to solids could be observed and analyzed reliably only for particles with a maximal size of ca. 20 μm. For bigger particles the potential binding of cells to solids, as analyzed by light microscopy was obscured due to the fact that cells no longer seen as free swimming might have bound to the particle, but also simply could be hidden in cavities of the solids being not transparent to light. Furthermore big particles did not allow to follow the tracks of swimming cells via high temperature video light microscopy; only the presence of a few particles per field of view allowed successful analyses. Bigger particles in addition resulted in problems with filling of the rectangular glass capillaries which have an inner height of 100 μm. It is for all these reasons that the data presented below show binding of cells to particles of a size between ~3 μm to a maximum of ~ 20 μm. The initial lava experiments also indicated how long a suspension of the solids (lava or BSM) should be standing after extensive shaking, allowing bigger particles to sediment, to withdraw via a syringe a suspension containing particles of the right size.

**Figure 1:**
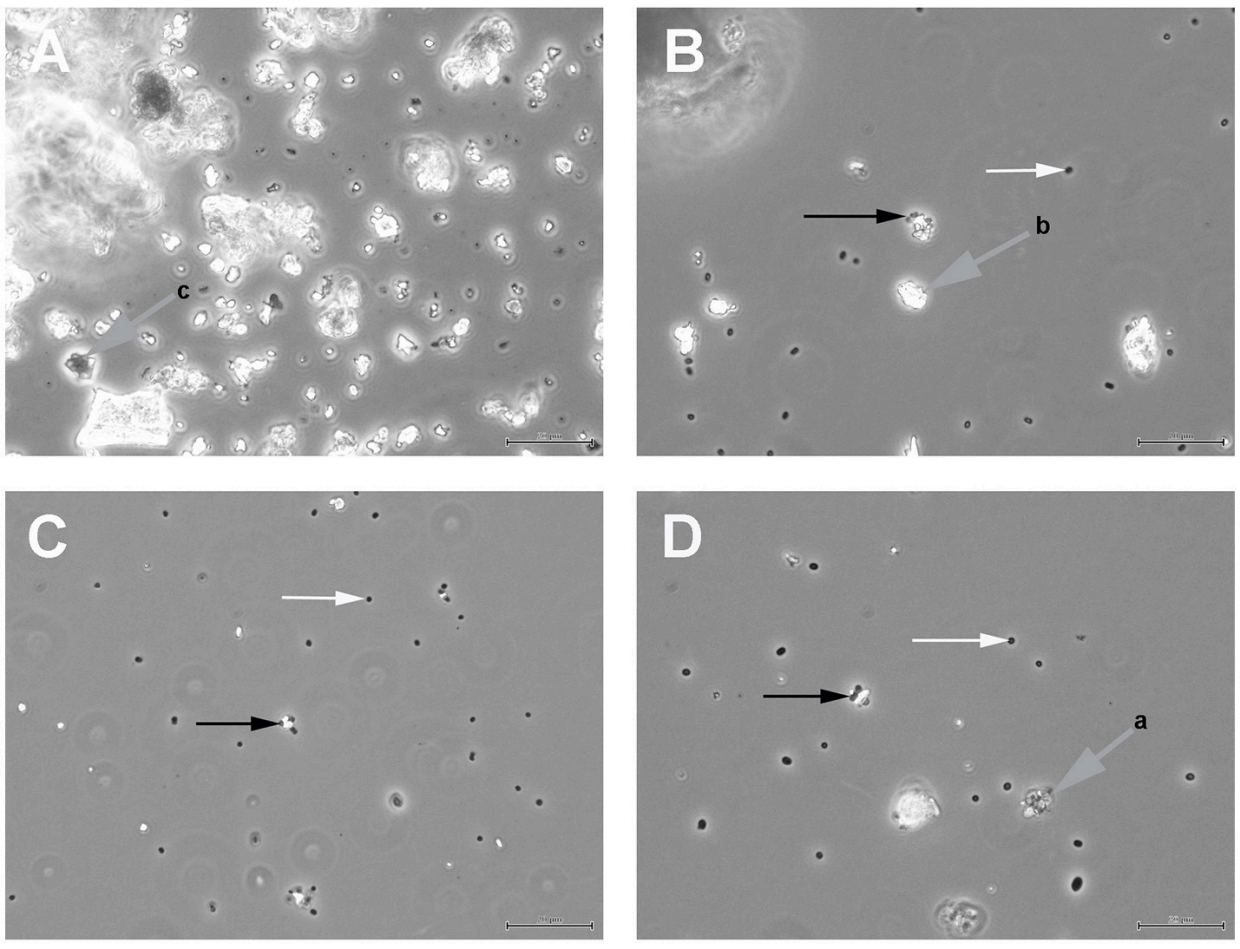
Light microscopic analyses of **(A)** the coarse suspension of lava from which the fines used for subsequent binding studies was derived; **(B)** *P. furiosus* strain BBR binding to the lava particles; **(C)** *P. furiosus* strain BBR binding to BSM; **(D)** *P. furiosus* strain Vc1 binding to BSM. Size bars: 50 μm for **(A)**; 20 μm for **(B–D)**. White arrows indicate free cells; black arrows indicate particles with adherent cells; gray arrows indicate different types of solids as identified by phase-contrast light microscopy.

**Figure 2:**
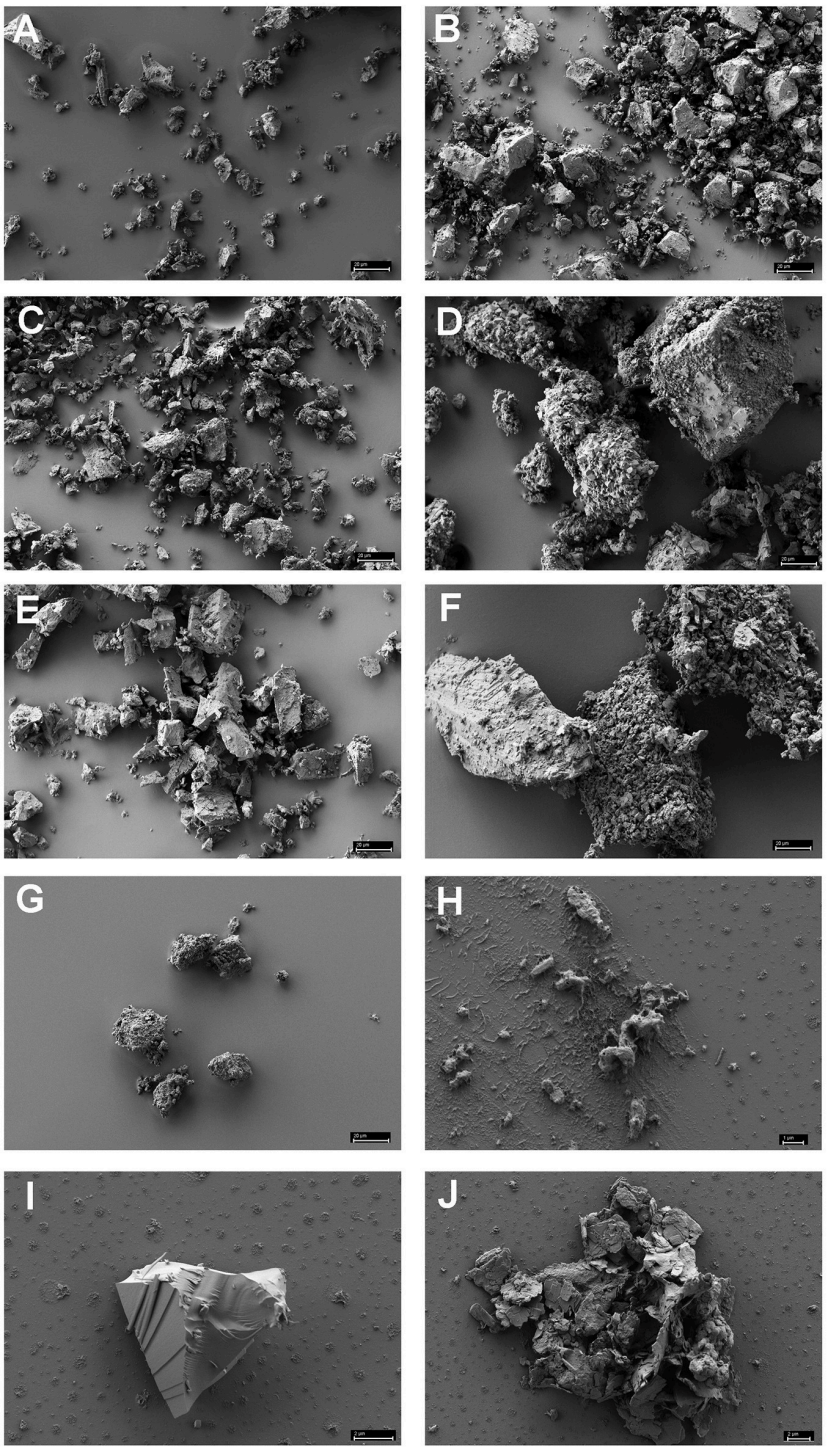
Scanning electron microscopy of various minerals reported to be often found in black smokers **(A–G)** and of authentic black smoker material **(H–J)**. **(A)** chalcopyrite; **(B)** hematite; **(C)** chalcosine; **(D)** pyrite; **(E)** sphalerite; **(F)** pyrite + sphalerite (1:1 in weight); **(G)** wurztite; **(H)** authentic BSM of the grainy, amorphous type a; **(I)**: authentic BSM with regular surface, type c; **(J)**: authentic BSM of the unstructured type b. Size bars in **(A–G)** 20 μm; in **(H)** 1 μm; in **(I,J)** 2 μm.

Figure 1 shows the results of such binding studies, analyzed by phase-contrast light microscopy; white arrows in Figures 1B–D indicate free cells, whilst black arrows point to solids with attached cells. Figure 1A shows the coarse suspension of lava from which the fines were withdrawn to be used for subsequent binding studies (without addition of cells). Figure 1B indicates that binding of *P. furiosus* strain BBR clearly takes place to these small lava particles and Figure 1C proves binding of this strain to BSM. Figure 1D finally shows that also *P. furiosus* strain Vc1 binds to BSM (see section Discussion for the binding behavior of various *P. furiosus* strains to surfaces). The fact that the pictures presented in Figure 1 do not show a mere occasional association between solids and cells was proven by the fact that we were able to sediment solids plus adherent cells, to suspend them thereafter in medium and cells still were attached; i.e., washing the solids did not remove cells. Via light microscopy we could differentiate between three types of solids: those with a very pronounced grainy structure (gray to black appearance by light microscopy—labeled with gray arrow a in Figure 1D); solids of irregular shape (very bright by light microscopy—labeled with gray arrow b in Figure 1B); solids with a more or less regular surface (black by light microscopy—labeled with gray arrow c in Figure 1A). Subsequent SEM analyses clearly resolved also these three structures; see Figure 2H, representing type a, Figure 2I, representing type c, and Figure 2J, representing type b. Via light microscopy only binding to solids of type a and b was observed; attachment to the c-type solids (Figure 2I) was an absolute exception.

Controls for such binding studies were experiments in which crushed cover glass and crushed quartz were present in growing *P. furiosus* cultures. After 3 h of incubation no adhering cells were detected, but after overnight incubation adhesion was observed to quartz, but not to cover glass (confirming the data reported in Table 1 of, Wirth, 2017). Adhesion to BSM and pure minerals typical for black smokers then is specific.

### Analyzing the binding behavior to BSM by high temperature video light microscopy

The BSM used for our studies had been collected in 2008; in 2013 “fines” were prepared from a part of sample MEX13C (such fines were subsequently used successfully as a source of potentially needed micronutrients in enrichment experiments). The autoclaved BSM fines were used in the studies reported here to ask if we could observe directly the binding event by taking videos at 95°C (*P. furiosus*) or 80°C (*M. villosus*). It turned out that we had to analyze over one hundred of videos to obtain reliable data. The main problem was that binding events as such were rare. A further complication came from the fact that not all initial binding events were stable; sometimes cells were attaching to the solids only for a few seconds, thereafter swimming away. Therefore only binding events in which the cells could be analyzed for at least 20 s to be stably attached to solids were counted as positive. In addition only binding events in which cells were attaching in the focus plane could be counted as positive. Events in which cells seemingly were binding to the solids somewhat outside of the focus plane and were not “reappearing” as swimming cells could not be counted as positive. A short description of the results of all video analyses is given below; we recommend the readers very much to actually look at the supplemental videos to understand these difficulties and especially to verify the following statements:

- Binding to the solids is preceded by a “scanning motion”; cells sometimes are “trembling” for up to 30 s at a distance of 1–3 μm from a particle before finally attaching to it—see Supplemental Movie 1.
- Direct binding—i.e., without the scanning motion—is with <3% a very rare event; Supplemental Movie 2 shows such an event, with the arrow pointing to the adhesion position.
- Not each scanning event followed by binding is permanent—see Supplemental Movie 3.
- Binding is not influenced by a fluid current—see Supplemental Movie 4. Here, the capillary was intentionally not sealed completely, resulting in some evaporation of medium, creating the fluid current.
- Cells not only can bind to solids; cells also can bind to cells already attached to solids—see Supplemental Movie 5.
- Cells can bind to small solids and then move these particles around—see Supplemental Movies 6, 7.

We did not observe a striking difference for the efficiency with which the two Archaea bound to the solids; i.e., *P. furiosus* and *M. villosus* seem to bind equally well to lava and to BSM. Finally we want to stress here again that the cells observed in our “binding movies” are close to a surface—the bottom of the glass capillary—and therefore nearly all of them are moving in the slow scanning mode. For comparisons cells of *M. villosus* swimming in the rapid translocation mode (focus plane is in the middle of the glass capillary) are shown in Supplemental Movie 8.

### Analyses of cells bound to BSM via SEM

**Figure 3:**
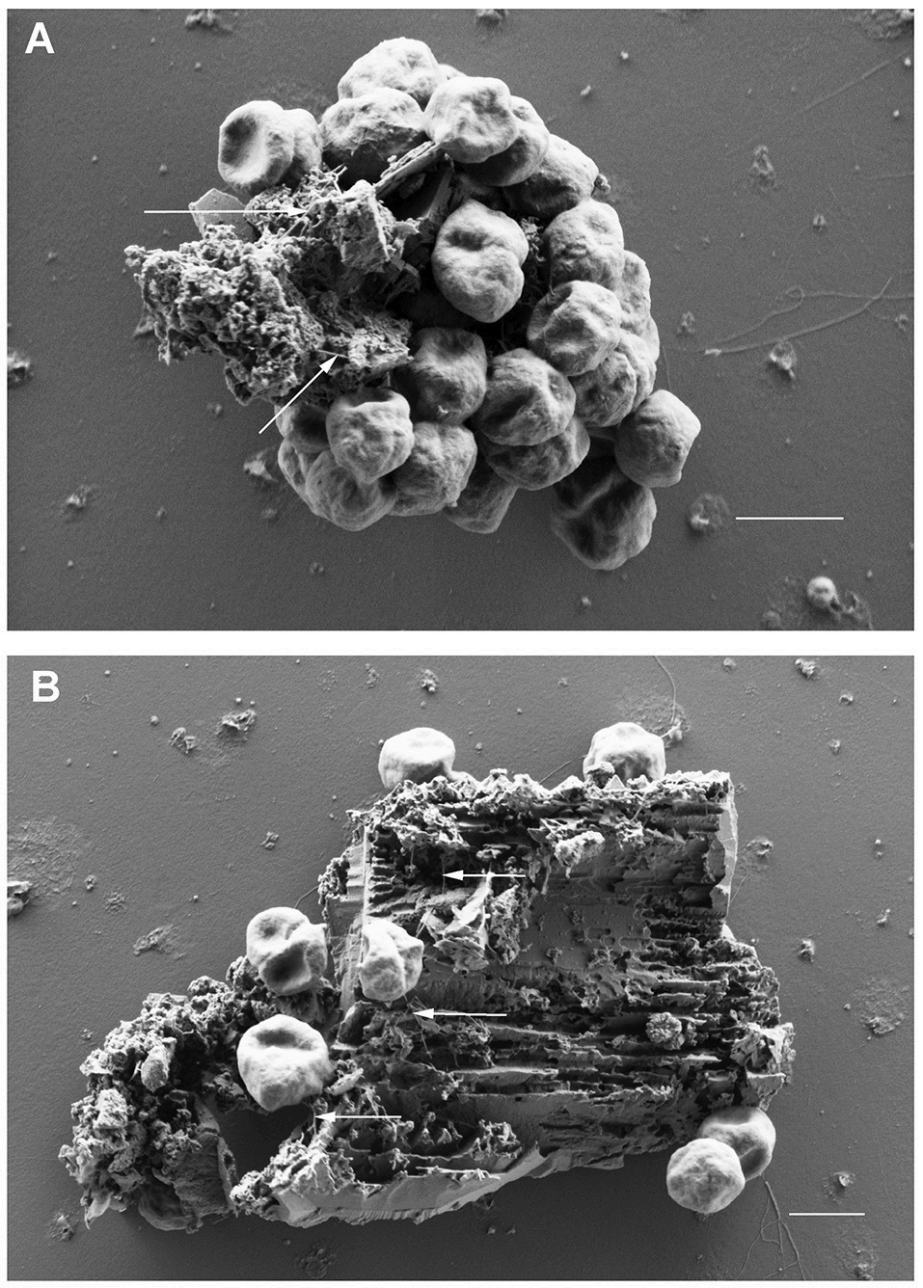
Scanning electron microscopic analyses of *P. furiosus* Vc1 cells adhering to BSM solids. **(A)** BSM solids of the grainy, amorphous type; **(B)** BSM solids of the unstructured type. Arrows in panels **(A,B)** highlight flagella. Size bars in **(A,B)** 1 μm.

Scanning electron microscopy was used to analyze the adherence of cells to small BSM particles in detail. BSM particles of the three types a to c identified by light microscopy were also detected by scanning electron microscopy: the grainy structured solids of type a are shown in Figure 2H; Figure 2I shows solids of type c with a more or less regular surface; type b solids with irregular shape as observed by light microscopy are exemplified by Figure 2J. Both, light microscopy and SEM analyses indicated that binding to solids with a regular surface is very seldom observed. Figures 3A,B show the results of binding assays of *P. furiosus* strain Vc1 to small BSM solids of types a, and b, respectively; interestingly type b exhibited a layered structure, only detectable by electron microscopy. Flagella binding to the surfaces are indicated by arrows in Figures 3A,B.

The identification of flagella was not easy in such binding experiments; their presence was obscured especially if many cells were bound to one particle. For single adhering cells at least one flagellum per cell could be identified—in most cases, however only over a short length of <1 μm. 3D anaglyph pictures (data not shown) indicated that the flagella were “inserted” into fissures, cracks, and slits of the solids which especially clearly are visible in Figure 3B. Our notion that the cells seen in Figures 3A,B really are actively adhering and not only passively bound to the particles is supported by the experimental procedure used for preparation of the samples. Solids (plus adhering cells) were in a first step collected by very mild centrifugation, removing >99% of free cells. Only after this first enrichment glutaraldehyde was added to those samples for fixation.

### Binding of cells to minerals found in black smokers

Since we had not observed adherence of cells to type c solids with regular surfaces, but to type a and b solids (exhibiting a structured surface as identified by light microscopy) it was of interest to ask to which type of minerals, typically found in black smokers, the two Archaea would bind. Seven different minerals were used for such binding studies; analyses were by light microscopy—the data are summarized in Table 1. The minerals chalcopyrite, hematite, sphalerite, and pyrite + sphalerite (1:1) were added in a final concentration of 0.25%. In the case of chalcosine, pyrite, and wurtzite this concentration inhibited growth of both Archaea. Reducing the concentration of these three minerals to 0.05% led to no delayed growth for pyrite and chalcosine and the concentration of the solids made observation of binding still possible. In the case of wurtzite even 0.025% inhibited growth and therefore no data could be collected for this β-ZnS. As Table 1 shows binding was observed to all minerals, but to different extents, with wurtzite not allowing statements on binding.

**Table 1:** Binding of *M. villosus* and *P. furiosus* Vc1 to the various minerals analyzed in Figures 3A–H reported to occur in black smokers.

	**Chalcopyrite CuFeS_2_**	**Hematite Fe_2_O_3_**	**Chalcosine CuS_2_**	**Pyrite FeS_2_**	**Sphalerite α-ZnS (Zn/Fe)S**	**Pyrite + Sphalerite (1:1)**	**Wurtzite β-ZnS**
*Pyrococcus furiosus* Vc1	0.25% ++ (delayed)	0.25% +++	0.25% inhibits growth, but 0.025% works +++	0.25% inhibits growth, but 0.025% works ++	0.25% +++	0.25% ++ (delayed)	0.25 and 0.025% inhibit growth!
*Methano-caldococcus villosus* KIN24	0.25% +++	0.25% +++	0.25% inhibits growth, but 0.025% works ++	0.25% inhibits growth, but 0.025% works +	0.25% +++	0.25% ++ (delayed)	0.25 and 0.025% inhibit growth!

*Binding was analyzed by light microscopy 1.5 and 3 h after addition of the minerals (in the indicated final concentrations; w/v) to cells in exponential growth phase. +++, > 20% of the solids show attached cells; ++, 2–10% of the solids have cells attached; +, ca. 1–2% of the solids have cells attached; delayed adhesion: definitely positive only after 3 h*.

SEM analyses of these different minerals indicated that none of them directly resembled the authentic BSM used for binding studies. The three-dimensional appearance of the minerals shown in Figures 3A,B are not completely congruent with those shown in Figures 2H–J, comprising authentic BSM. We therefore cannot argue to which materials or their components the Archaea tested here do bind best.

## Discussion

It had been postulated recently that *Colonization of a newly erupted black smoker occurs by a first accidental contact of hyperthermophiles present in cold seawater. The cells react within seconds to the high temperature present at the surface, by very fast motility, and swim to a region whose temperature is optimal for them. Via their much slower zigzag mode of swimming, they scan for a place optimal for adhesion and use their motility organelle to establish contact. Their flagella allow them a first attachment and growth in mono- and at least bi-species biofilms there; additional adhesins well might contribute to permanent colonization (Wirth*, *2017**)*. This scenario was formulated based on various data from our labs; we especially had shown earlier that:

- Hyperthermophiles can react within a maximum of 5 s to high temperature by starting motility (Mora et al., 2014).
- Hyperthermophiles are extremely fast swimmers (Herzog and Wirth, 2012).
- Hyperthermophiles have two swimming modes, one for rapid translocation over big distances and a much slower zigzag movement to scan surfaces (Herzog and Wirth, 2012).
- The two model organisms used here are able to adhere onto various surfaces via their flagella (Näther et al., 2006; Bellack et al., 2011). Data for adhesion of 7 hyperthermophiles, including the two species used here, to a total of 22 materials were summarized recently (Wirth, 2017—Table 1 in Supplemental Information).

We deliberately have chosen to use *P. furiosus* and *M. villosus* as study objects because they are often used model organisms for hyperthermophiles, especially if asking for motility and adhesion (Näther et al., 2006; Bellack et al., 2011; Herzog and Wirth, 2012; Mora et al., 2014; Näther-Schindler et al., 2014). In addition, they are representatives of Thermococcales *and* Methanococcales, which were reported to be the most abundant Euryarchaeota in Z3 of Finn, the black smoker analyzed in detail for the presence of microorganisms in an active sulfide chimney (Schrenk et al., 2003). In the case of *P. furiosus* we used the three strains Vc1, BBR and LS for our studies, because they differ especially in their number of flagella and adherence to various surfaces (Bellack, 2011; Näther-Schindler et al., 2014). Since no striking differences were observed in our first experiments, studying adhesion to BSM, all further analyses were only done using strain Vc1. This is the original strain isolated off the Coast of Volcano Island (Italy; Fiala and Stetter, 1986), and therefore it is the best choice for a wildtype isolate.

A variety of other studies exist which were asking for the presence of hyperthermophiles in black smokers (see Wirth, 2017 for a detailed discussion). Two important studies were asking for primary colonization events (McCliment et al., 2006; Pagé et al., 2008). McCliment et al. removed actively venting chimneys during two expeditions to the East Pacific Rise in 2001 and 2002 and deployed mineral chambers and sampling units that allowed growth of new chimneys (McCliment et al., 2006). The new chimneys were collected within 72–92 h and analyzed for microorganisms by 16S rDNA analyses (the old and new chimneys also were analyzed with respect to temperature and minerals). It turned out that under these conditions mainly members of the genus Ignicoccus and its symbiont Nanaoarchaeum were detected. In 2003 Page et al. deployed thermocouple arrays into a knocked over mushroom structure at Guaymas Basin, Mexico and collected these deployments after 4 and 72 days (Pagé et al., 2008). The thermocouples were overgrown with mineral deposits from the hydrothermal vents and subsamples there from were analyzed for archaeal diversity via 16S rRNA gene analyses. No indication for hyperthermophiles was found at regions >200°C, but distinct phylotypes were detected for regions of 110°C (*Methanocaldococcus* spp.) and 116°C (Desulfurococcaceae). It was argued in this latter study that a temporal transition in the carbon sources occurred in these new forming deposits, reflected by a transition of the archaeal communities. Both of these studies, therefore show that colonization occurs within days, but could not provide any data as for the actual primary events, especially binding of hyperthermophiles to authentic minerals of hydrothermal vents.

We have shown here that the two Archaea *P. furiosus* and *M. villosus*—often used model organisms for hyperthermophiles—are able to attach to solids from lava, but especially also from an authentic black smoker. This adhesion was proven by light and electron microscopy and we have been able especially to directly analyze the very first attachment process by high temperature light microscopy. We note that the identification of flagella by SEM was not as easy and clear in the experiments reported here as it was for binding assays of *P. furiosus* to e.g., sand grains (Näther et al., 2006) and of *M. villosus* to e.g., carbon on grids used for electron microscopy (Bellack et al., 2011). We could show that this comes from the fact that the assays with BSM fines led to some “background” in the SEM pictures, caused by material “leaking” from BSM—compare backgrounds in Figures 2H–J with that in Figure 2A–G. Assays in which we added carbon coated grids for electron microscopy into BSM containing binding experiments revealed that cells attaching onto the carbon also were coated with some background material obscuring identification of flagella (data not shown). Nevertheless the data reported here in their entirety indicate that the scenario how hyperthermophiles colonize a newly formed black smoker is correct, but they also raise further questions.

(i) Our data show that the cells obviously are scanning the surface of solids by the earlier described zigzag movements and finally adhere tightly directly onto the surface. It is not known which mechanisms the cells use for their two different swimming modes: rapid translocation by very fast, ±linear swimming tracks vs. much slower zigzag movement close to surfaces. We want to note in this connection that both Archaea used here have ca. 50 flagella per cell inserted at one pole (Fiala and Stetter, 1986; Bellack et al., 2011). Our ideas by which mechanisms the two swimming modes are caused assume that rapid swimming is done by a concerted rotation of all flagella. The zigzag mode then might be caused by rotation of only some flagella, whilst non-rotating flagella could be used to scan a surface and attach to it [see also (ii) below]. This scenario is indirectly supported by our observation that cells can adhere to small solids and move those around (see Supplemental Movies 6, 7). Here, some flagella would bind the cells to the surface, whilst other flagella are rotating for movement. As for the moment there are no hard data supporting this speculation; one potential way to prove this scenario would be staining of flagella by fluorescent dyes and analyses of free swimming and zigzag swimming cells. This staining and analysis approach of living cells was developed initially for the model bacterium *Escherichia coli* (Turner et al., 2000), but seems in general not to work for Archaea—at least for 46 species including the two Archaea used here (Wirth et al., 2011).

(ii) As for the moment it is not clear by which mechanism(s) archaeal cells recognize solids onto which they adhere. Such mechanisms are in a few cases very well-understood for other microorganisms, especially pathogenic bacteria. In the latter case we do know that it is the tip of bacterial cell appendages interacting with surfaces of eukaryotic cells. In the case of P-pili the structure of the tip of the pilus, i.e., the pilin PapG binding to globotriasylceramide (the surface receptors of eukaryotic cells) is known down to molecular scale (Dodson et al., 2001; Sung et al., 2001). In case of the type 4 and type 6 secretion systems—T4SS and T6SS—their recognition of target receptors also is known in great details (Basler et al., 2012; Low et al., 2014; see Costa et al., 2015 for review). The extracellular structures used by various bacteria to recognize metal surfaces to be used for electron transfer (for “respiring stones”) also were and are analyzed intensively (see Shi et al., 2016 and Flemming et al., 2016 for reviews). In the case of archaeal cell appendages no data are available by which mechanism(s?) they recognize surfaces with which they interact.

If archaeal flagella do have different flagellins distributed not evenly over their length is not known in detail—especially we do not know if one flagellin is located only at the flagellar tip, and thereby could be used as receptor for surfaces. In the case of *Methanococcus voltae* the minor flagellin FlaB3 has been reported to be located in a region of the flagellum equivalent to the hook region of bacterial flagella (Bardy et al., 2002), i.e., at the base rather than at the tip. Our previous data indicated that the minor flagellins FlaB1 and FlaB2 of *P. furiosus* flagella probably are located on one end of the structure, but it is not known if this end represents the base or the tip of the motility organelle (Näther-Schindler et al., 2014). In addition ultrastructural analyses (Daum et al., 2017) show that the main part of the *P. furiosus* flagellum filament is composed exclusively by the major flagellin FlaB0. This then indicates that the minor flagellins FlaB1 and FlaB2 might be located at “end regions” of the flagellum, but again do not define these regions as base or tip of the archaeal flagellum. Therefore, further experiments should ask for the distribution of archaeal flagellins over the length of this motility organelle. It is interesting in this respect that the existence of more than one flagellin gene is the rule, and not an exception for Archaea, standing in contrast to Bacteria (Jarrell and Albers, 2012; Albers and Jarrell, 2015). This also holds true for the two species used here, both of which possess three flagellin genes (Thennarasu et al., 2013; Näther-Schindler et al., 2014).

(iii) Our movies of swimming Archaea indicate that they are scanning the surface and finally adhere directly to it—i.e., without a visible distance between cell and surface. This final step in the adhesion process is a fast one, requiring in maximum 1–3 s. This then raises the question if we observe here a motion process similar to that mediated by type IV pili of Bacteria. The so-called twitching motility of Bacteria involves the rapid depolymerization of their type IV pilins into the cytoplasmic membrane within 1–2 s (see e.g., Burrows, 2012 for review). Archaeal flagella have been defined as one component of many type IV pilin-like structures found in Archaea (Albers and Pohlschröder, 2009). It has to be noted in this connection that the definition for bacterial type IV pili and archaeal type IV pili differ: the first ones exclusively are pili used for twitching motility; the latter ones simply are defined by a similar N-terminal region of the respective protein, but not by its function and therefore contain many structures with different functions, like pili, flagella, S-layers (Makarova et al., 2016). The conserved N-terminus defining the archaeal type IV pili structures has been shown recently to form an α-helix located in the interior part of the respective structure; it functions as a backbone for archaeal flagella (Yu et al., 2012; Braun et al., 2016; Poweleit et al., 2016; Daum et al., 2017). Our observation that the final adhesion step is a fast one could be interpreted in the way that at least the two Archaea analyzed here might use the bacterial type IV typical twitching mode to pull the cells to the surface. Clearly further experimentation is warranted to ask if this interpretation of our observations is correct.

(iv) The BSM used in this study is derived from one black smoker, sampled at the East Pacific Rise. To fit into the rectangular glass capillaries (100 μm inner height) used for our video analyses, BSM had to be ground to a state that the solids had a size of maximal 20 μm. This then means that we used a mixture of various regions of the black smoker; but various regions of black smokers have a different mineralogy. In the case of black smoker Finn the following composition has been determined (Schrenk et al., 2003): Z1 was composed of mainly oxidized sulfide minerals; Z2 contained iron- and zinc-sulfide minerals together with amorphous silica, in addition some barite, anhydrite and clay minerals were locally present; Z3 contained predominantly pyrite and zinc sulfide, together with chalcopyrite and local patches of anhydrite; Z4 finally consisted mainly of chalcopyrite plus some zinc sulfide. In addition it has to be stressed, that “THE” Black Smoker does not exist; the vent fluids from one hydrothermal field forming the chimneys can vary in their composition not only by their location, but even over time (Kelley et al., 2002). We therefore have chosen minerals most often reported to occur in black smokers (Haymon, 1983; Tivey, 1998; Holden et al., 2012) to ask onto which of these different defined minerals the two Archaea would attach. These minerals were:

- Chalcopyrite, mostly found as inner lining of mature black smokers.
- Hematite, present as inclusion in black smoker derived chalcopyrite.
- Chalcosine, mostly found in middle layers of mature black smokers.
- Pyrite, mostly found in middle to outer layers of new forming and mature black smokers.
- Sphalerite, mostly found in outermost layers of new forming and mature black smokers.
- Pyrite/sphalerite in a 1:1 ratio.
- Wurtzite, mostly present in vent fluids, but also as inclusion in chalcopyrite.

Of these minerals wurtzite inhibited growth of both Archaea even in a concentration of 0.025% which was the lowest concentration to be used for binding assays analyzed by phase-contrast light microscopy. For all other minerals we did observe clearly adhesion to mineral particles, very nicely supporting our data for authentic BSM. SEM analyses of the 7 minerals indicated that they have not the same structure than the mortar crushed BSM used here (compare Figures 2A–G with Figures 2H–J. One reason for this might be the fact that during formation of black smokers intensive mineralogical rearrangements take place (see Haymon, 1983 for review and references therein and Tivey, 1998) and therefore authentic BSM material very well might not be composed of just one of the pure minerals used by us. This indeed seems to be the case: EDX analyses (data not shown) indicate that our BSM preparation consists mainly of pyrite; different BSM particles contain varying, but small amounts of silicium, sodium, zinc, and copper, too. We want to state that it would “make sense” in our eyes for Archaea to be able to adhere to a variety of minerals occurring in black smokers, because the distribution of such minerals within the porous wall is varying over time with development of the chimneys (Haymon, 1983; Tivey, 1998). The distribution of Archaea and Bacteria within certain layers of black smokers indeed is varying to a great extent (Takai et al., 2001; Schrenk et al., 2003). Therefore also the data obtained with binding of archaeal cells to minerals typically found in black smokers support the scenario for colonization of black smokers by hyperthermophiles as stated earlier (Wirth, 2017).

In summary: no hard (= experimental) data exist for the mechanism of colonization of newly erupted, sterile black smokers by hyperthermophiles. Such experiments had to be done with newly forming vent chimneys. In our eyes it is at least questionable that one should be able to sample wall material (probably by coring) under strictly sterile conditions, without inclusion of background seawater, from the top of a newly forming black smoker, to return repeatedly to the same black smoker within a few hours to days (colonization is a rapid process—see above and Wirth, 2017 for details) and to take samples from the same spot of that vent chimney asking for the presence of live hyperthermophiles and their cell surface appendages in these samples. The approach used here, namely asking for adhesion properties of hyperthermophiles onto authentic BSM, then is the best validation (we can think of) for the hypothesis how black smokers are colonized by hyperthermophiles.

## Author contributions

RW designed the study and performed research using light microscopy. ML and GW made all electron microscopic analyses. RW and GW wrote the paper. All authors agreed to the final version.

### Conflict of interest statement

The authors declare that the research was conducted in the absence of any commercial or financial relationships that could be construed as a potential conflict of interest.

## Supplementary material

Supplemental Movie 1 Adhesion of *Pyrococcus furiosus* onto lava particle after scanning the surface.

Supplemental Movie 2 Rapid adhesion of *Methanocaldococcus villosus* onto black smoker particle. The arrow indicates the point of adhesion.

Supplemental Movie 3 Short term binding of *Methanocaldococcus villosus* onto black smoker particle followed by detachment.

Supplemental Movie 4 Adhesion of *Methanocaldococcus villosus* onto black smoker particle, independent from fluid current.

Supplemental Movie 5 Binding of *Pyrococcus furiosus* onto another *P. furiosus* cell, itself attached to a black smoker particle.

Supplemental Movie 6 Cells of *Methanocaldococcus villosus* can adhere onto a small black smoker particle and move that around.

Supplemental Movie 7 Cells of *Pyrococcus furiosus* can adhere onto lava particles and move those around.

Supplemental Movie 8 Motility of free swimming *Methanocaldococcus villosus*.

Supplemental Movies 1–6 show sections of the complete screen, using a two-fold secondary magnification; Movies 7 and 8 show the complete screen.
